# Computational modeling of the obstructive lung diseases asthma and COPD

**DOI:** 10.1186/1479-5876-12-S2-S5

**Published:** 2014-11-28

**Authors:** Kelly Suzanne Burrowes, Tom Doel, Chris Brightling

**Affiliations:** 1 Department of Computer Science, University of Oxford, Oxford, UK; 2 Institute for Lung Health, Department of Infection, Immunity and Inflammation, University Hospitals of Leicester, Leicester, UK

## Abstract

Asthma and chronic obstructive pulmonary disease (COPD) are characterized by airway obstruction and airflow limitation and pose a huge burden to society. These obstructive lung diseases impact the lung physiology across multiple biological scales. Environmental stimuli are introduced via inhalation at the organ scale, and consequently impact upon the tissue, cellular and sub-cellular scale by triggering signaling pathways. These changes are propagated upwards to the organ level again and vice versa. In order to understand the pathophysiology behind these diseases we need to integrate and understand changes occurring across these scales and this is the driving force for multiscale computational modeling.

There is an urgent need for improved diagnosis and assessment of obstructive lung diseases. Standard clinical measures are based on global function tests which ignore the highly heterogeneous regional changes that are characteristic of obstructive lung disease pathophysiology. Advances in scanning technology such as hyperpolarized gas MRI has led to new regional measurements of ventilation, perfusion and gas diffusion in the lungs, while new image processing techniques allow these measures to be combined with information from structural imaging such as Computed Tomography (CT). However, it is not yet known how to derive clinical measures for obstructive diseases from this wealth of new data. Computational modeling offers a powerful approach for investigating this relationship between imaging measurements and disease severity, and understanding the effects of different disease subtypes, which is key to developing improved diagnostic methods.

Gaining an understanding of a system as complex as the respiratory system is difficult if not impossible via experimental methods alone. Computational models offer a complementary method to unravel the structure-function relationships occurring within a multiscale, multiphysics system such as this. Here we review the current state-of-the-art in techniques developed for pulmonary image analysis, development of structural models of the respiratory system and predictions of function within these models. We discuss application of modeling techniques to obstructive lung diseases, namely asthma and emphysema and the use of models to predict response to therapy. Finally we introduce a large European project, AirPROM that is developing multiscale models to investigate structure-function relationships in asthma and COPD.

## Introduction

Chronic obstructive lung diseases, the most prominent being that of asthma and chronic obstructive pulmonary disease (COPD), are common and exert a large burden on society [1]. These diseases affect over 500 million people worldwide [2] with related costs exceeding €56 billion per year in the European Union. These diseases are characterized by an underlying inflammatory response that encompass pathophysiological changes across a range of biological - spatial and time - scales: from gene to cell, tissue to whole organ. The diminished lung function in obstructive lung diseases is characterized by an underlying inflammatory response that induces airway remodeling, airflow limitation and increased ventilation-perfusion (V/Q) mismatch. Whilst the hallmark of asthma is variable airflow obstruction and airway hyper-responsiveness of the airway smooth muscle (ASM), COPD is characterized by permanent and irreversible airflow obstruction. Capturing the physics of this system requires detailed structural models as well as models predicting alterations in mechanical properties of the tissue during disease and the dynamics of fluid flows. It has been realized that the emergent function of the lung cannot necessarily be predicted using a reductionist approach [3]: that is, resulting function may not be predicted realistically by considering the component parts in isolation. It is the nonlinear interaction between the parts that leads to unexpected properties; hence the need for the development of multiscale models.

The Physiome project (http://physiomeproject.org) started in 1997 with the aim of developing a framework for modeling the entire human body. The Virtual Physiological Human initiative (http://www.vph-institute.org) followed not long after with similar goals but with a particular emphasis on personalized medicine. These initiatives ultimately aim to bridge the gaps in physiology - bringing together data from disparate scales of biology and providing a means of interpreting or inferring alterations in regional properties across the biological scales from molecule to organ. Technical areas being addressed around the world as part of these initiatives include data handling, markup languages, model repositories, ontologies, computational tools as well as development of grid, middleware, and workflows [4]. Probably the most developed Physiome/VPH-type model is that of the heart. For example, cardiac electrophysiology models have been used to computationally assess drug-induced effects on the electrocardiogram; with models linking right from ion-channels to predictions of body surface potential [5]. Models of the respiratory system are following closely behind developments such as this with, for example, personalized models of airflow used to predict the response to therapy [6-9] and multiscale models of ventilation in the asthmatic lung [3,10,11].

The diagnosis and severity classifications of obstructive lung diseases are largely based on global lung measurements from pulmonary function tests (PFTs). However these measures are insufficient for current diagnostic needs because, although they can be used to attribute future risk of disease progression, future exacerbations and mortality in populations, they are poor predictors of patient outcomes for individuals [12]. PFTs provide measurements only of global lung function and cannot detect the regional functional changes characteristic of lung diseases. It is these heterogeneities that we need to characterize on a patient-specific basis in order to advance healthcare. For this reason medical imaging is increasingly being used in the diagnosis of lung disease, however small airways are not visible with current imaging modalities. For this reason a combined imaging and modeling approach is required to obtain novel information about the respiratory system. Imaging and image processing techniques are critical in the construction, parameterization and validation of patient-specific computational models. In this review, we begin by outlining current state-of-the-art tools and techniques related to computational modeling of the lung and pulmonary image analysis. We describe how models have so far been applied to understanding or improving analysis related to obstructive lung diseases and in providing more sensitive measures of response to therapy. We finish by outlining the work in an exemplar lung modeling project, AirPROM.

## Computational modeling of the lung

### Structural models of the lung

Early models of the lung utilized generic representations of the structure - for example, using symmetrically branching networks to represent airways and vessels [13]. Rapid advancement in imaging and image processing techniques, described below, has enabled the relatively straightforward generation of anatomically based patient-specific models possible. This is important as both experimental and simulation studies have demonstrated the influence of geometric structure on functional outcomes [14].

State-of-the-art structural models used for 3D CFD are now fully patient specific consisting of high-resolution computational meshes of the central airways (typically including 7-9 generations of branching) containing in the order of millions of computational elements [15,16]. Smaller airways however are not currently represented in these types of models due to computational and time restrictions on the size of the meshes. A simplified 1D approach is necessary to represent the structure of the full airway and vascular networks. Tawhai et al. [17] developed a volume-filling branching (VFB) algorithm, which has provided a morphometrically-realistic method to generate patient-specific network models, used in several modeling studies [11,18-24]. This method generates branches from the end points of airways/vessels derived from high-resolution images (typically CT images at full inspiration) and grows a branching tree into the lobar volumes also derived from imaging. Note that the central airways and lobar volumes and shapes are patient-specific while the additional 1D branches are only an estimation of the structure (but are realistic according to published branching statistics) of the distal airway tree. 1D models require information regarding branch diameter to be allocated and this is normally derived from the visible CT branches plus morphometric information published in the literature.

### Parameterizing models from imaging

Both structural and functional information can be extracted from imaging and used to parameterize or validate computational models. Structural information is becoming relatively well established in the modeling pipeline. CT is the modality of choice for structural lung imaging due to its high spatial resolution and good signal-to-noise ratio [25]. The CT Hounsfield Unit (HU) provides a measure of density and enables extraction of structural features such as the geometry of airways, vessels and lobes, vital for the development of personalized models.

Airway segmentation is commonly performed using threshold-based region-growing methods to detect the airway lumen [25]. The centerline can be determined using local properties of the image or skeletonization. The computational mesh of the airway segmentation may be used directly in 3D CFD simulations, while the centerline and radius information are used in the development of personalized 1D functional models. The wall thickness can be extracted and used to assess inflammation effects of disease. Vessel segmentation has been performed using Hessian-based approaches such as the vesselness filter [26].

**Figure 1:**
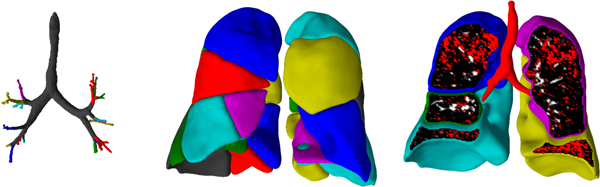
**Combined image-processing and modeling results from a CT scan of a patient with COPD from the Synergy-COPD project**. Segmentation of (A) central airways and (B) segments (calculated using both image processing and modeling approaches, color coded by segment). (C) Displays emphysematous regions (emphysema indicated in white and normal tissue in brown) extracted using thresholding of the image intensity values (emphysema <950 HU) [Doel, 2014, unpublished results].

Lobe segmentation is typically performed by dividing the segmented lung volume through detection of the pulmonary fissures using various methods (see review by [25]). Some authors extend lobar segmentation ideas to extract the pulmonary segments (see review by [25]). However, validation is more difficult as the segment boundaries are not generally identifiable on CT scans. A novel technique combining imaging and modeling (via the VFB) has been used to extract pulmonary segments within the Synergy-COPD project (Figure 1, Doel, 2014, unpublished information). High resolution CT data used here was provided with permission by Hospital Clinic, IDIBAPS, University of Barcelona using a multislice spiral CT scanner (Somatom Sensation 64) as part of the Synergy-COPD project.

The regional assessment of obstructive disease can also be used to parameterize or validate computational models. A great deal of research has been carried out into automated assessment of COPD from CT images [27]. The amount of emphysema, bronchial wall thickening and air-trapping can all by quantified using CT. Emphysema is typically quantified by computing the percentage of voxels which fall below a simple threshold such as −950 HU.

Functional imaging offers a wealth of regional data for model parameterization and validation. In recent years, one of the biggest areas of development for functional imaging of the lung has been in hyperpolarized gas MR imaging (HP MRI) [28]. Measures of both static and dynamic ventilation are possible, providing information on the distribution of disease attributes such as ventilation defects and gas trapping [29,30]. Diffusion-weighted imaging measures the apparent diffusion coefficient (ADC) of the gas and provides a measure of the alveolar structure [31]. Increased ADC values indicate emphysema and a increased regional heterogeneity in ADC values have been found in both COPD and asthmatic subjects [32].

### Functional models of the lung

Models of several different aspects of respiratory function have been developed including models of ventilation [15,18,19,33], perfusion [20,23,34], gas exchange [35], forced expiration [36,37], gas washout [24,38], impedance [21,22], particle transport and deposition [16,39,40] and tissue mechanics [41,42]. Nearly all of these modeling studies have only investigated function in a normal healthy lung, with relatively few studies investigating obstructive lung diseases. In this section we focus our discussion on models representing disease and in particular multiscale models and studies that have combined imaging and modeling methodologies.

### Applications of models to bronchoconstriction/asthma

There is a paucity of truly multiscale respiratory models. An excellent example of a multiscale model investigating lung disease is that of Donovan et al. [10,11]. This work has investigated the impact of bronchoconstriction on lung function and links together events at the cellular and molecular level to function in the whole organ. This model includes patient-specific lobes and central airways and includes distal airways created using the VFB method. The airway model is embedded within a 'breathing' mechanics model. The tissue model for each airway is linked to a model of the cross bridge mechanics in the ASM and their control by calcium dynamics. This provides a link right across the scales and has most recently been used to demonstrate insight into the synergistic interaction between deep inspirations and ASM cell fluidization in reversing airway closure (linking organ level motion to cell and tissue level function) [11].

Another example is the model developed by Venegas et al. which incorporated an airway network, the distribution of airflow, airway wall mechanics and ASM behavior [3]. Only minimal heterogeneity was required in the model (in airway wall thickness) to cause a vicious cycle that produced a so-called catastrophic shift in airway structure producing the self-organized patchy ventilation observed in functional images of asthmatics. This behavior would not have been observed by the simple summation of model components, the system required interdependence between the parts to produce this emergent outcome, highlighting the necessity of having a fully integrated model to predict realistic outcomes.

Various other modeling studies have presented novel outcomes through a combined imaging and modeling approach [21,22]. These models incorporate patient-specific airway branching geometry and in addition prescribe functional defects from images. In general the structure of the models are manipulated in order to replicate the function observed in the images. This provides a way to infer structural properties of the small airways which cannot be measured via imaging. These studies have typically shown that ventilation defects observed in asthmatics must be caused by fairly severe constriction of small airways (less than 2 mm in diameter).

### Applications of models to COPD/emphysema

There have been far fewer modeling studies focusing on COPD, with most studies considering only emphysema. Models of emphysema have predominantly focused on the alterations occurring in the parenchymal tissue and the resultant change in tissue mechanical properties. The most common approach uses a network of springs to represent the lung parenchymal tissue [43,44], with individual springs representing alveolar walls. These modeling studies have highlighted the redistribution of forces within the tissue during the progression of emphysema. To initiate emphysema a random percentage of springs were removed. Additional springs under the highest tension were preferentially removed to mimic mechanical failure and emphysema progression. This method of progression has been found to produce patterns of emphysema observed experimentally [44] and provide important evidence for the role of mechanical forces in emphysema progression. This approach was extended to a larger network and revealed sudden changes in the macroscopic stiffness of the tissue when 'failed' springs connected in a continuous pathway across the network [43]. A similar study applied a 3D model, using cuboidal cells to represent alveoli, to investigate the relationship between the pattern of alveolar wall destruction and the emergent mechanical properties of the tissue [45]. This study demonstrated that the distribution of emphysematous lesions plays a vital role in the resultant organ level function. These types of studies - linking microscopic changes to macroscopic outcomes - are excellent examples of the added knowledge computational modeling can provide.

### Functional respiratory imaging to predict the response to therapy

Functional Respiratory Imaging (FRI) combines the computational modeling technique of 3D CFD with imaging to create patient-specific models of lung function. Pioneering studies by FLUIDDA, a small company based in Belgium, have applied this technique to provide a novel tool for phenotyping patients and monitoring the efficacy of novel respiratory drugs [7-9]. Results from over 20 clinical trials involving a total of 750 patients have demonstrated the higher sensitivity of FRI outcomes, in comparison with standard diagnostic measures such as spirometry, through its use of regional FRI biomarkers.

**Figure 2:**
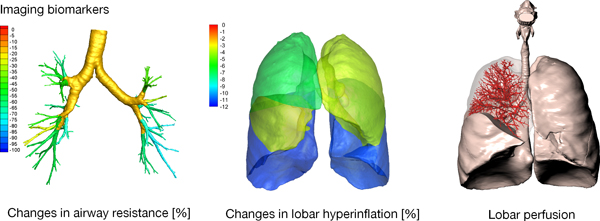
**Illustration of the typical biomarkers extracted by FLUIDDA's work which combines computational modeling with medical imaging to produce an approach termed Functional Respiratory Imaging (FRI) **[57]**Reproduced with permission from FluidDA**.

CT images at full inspiration and full expiration are required for this technique. Segmentation techniques are applied to extract the lobar volumes and central airway geometry. Patient-specific geometric models are created of the central airways and the patient-specific change in lobar volume from expiration to inspiration are applied in the CFD flow boundary conditions [15]. FRI is able to produce clinically relevant patient specific biomarkers such as the lobar and airway volumes, internal airflow distribution, airway resistance, lobar perfusion and regional aerosol deposition (see Figure 2).

## Multiscale models of asthma and COPD: the AirPROM approach

In this section we will discuss a large European-wide project called AirPROM (**Air**way disease **PR**edicting **O**utcomes through patient-specific computational **M**odeling; http://www.airprom.eu) [46]. This is an excellent exemplar of the development of multiscale models of the lung applied to obstructive lung disease that relates well to the Synergy-COPD study (discussed in this supplement [47]). The ultimate goal of AirPROM is to bring together data and computational models across disparate scales to develop an integrated multiscale model of the airways in order to unravel the pathophysiological mechanisms occurring in asthma and COPD. The modeling within this project is clinically driven pushing modeling developments in the direction of tangible clinical outputs. Two of the significant assets of AirPROM are the large amounts of well-characterized clinical data and the truly multidisciplinarity of the consortium with expertise in clinical medicine, physiology, radiology, image analysis, bioengineering, data handling, computational modeling and systems biology. The concept behind this work is to move away from the typical 'one size fits all' treatment of respiratory disease towards patient stratification and personalized medicine.

The clinical data consists of both asthmatic and COPD patients from aligned consortia EvA [48], UBIOPRED [49] and BTS Severe Asthma network [50]. These data include extensive genomic, transcriptomic and proteomic profiles, detailed lung function with novel small airway physiological measures [51], bronchial challenge studies, CT [52] and HP MRI imaging data, and patient-reported outcomes. The data provides both cross-sectional and longitudinal follow-up data including some clinical intervention studies. These data have been integrated into a data management platform that will feed into the computational modeling framework [53]. This is the same knowledge management framework developed and used within the Synergy-COPD project.

**Figure 3:**
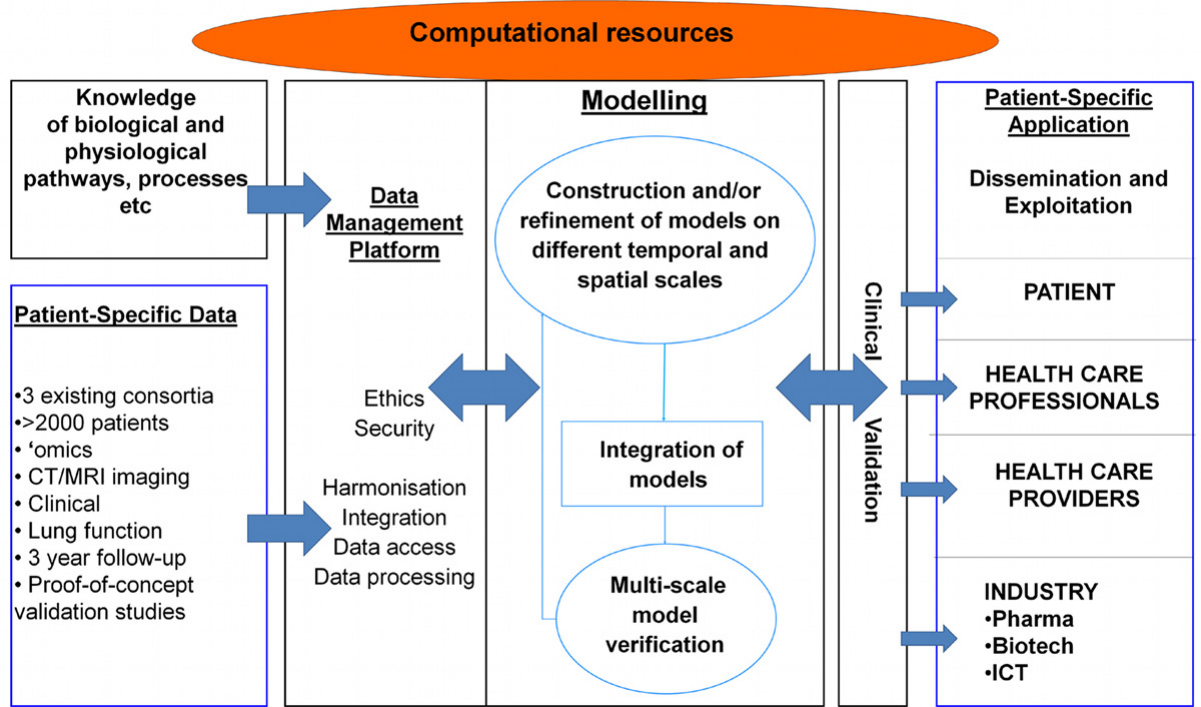
**Schematic diagram illustrating the workflow structure within the AirPROM project**. Figure courtesy of [46].

**Figure 4:**
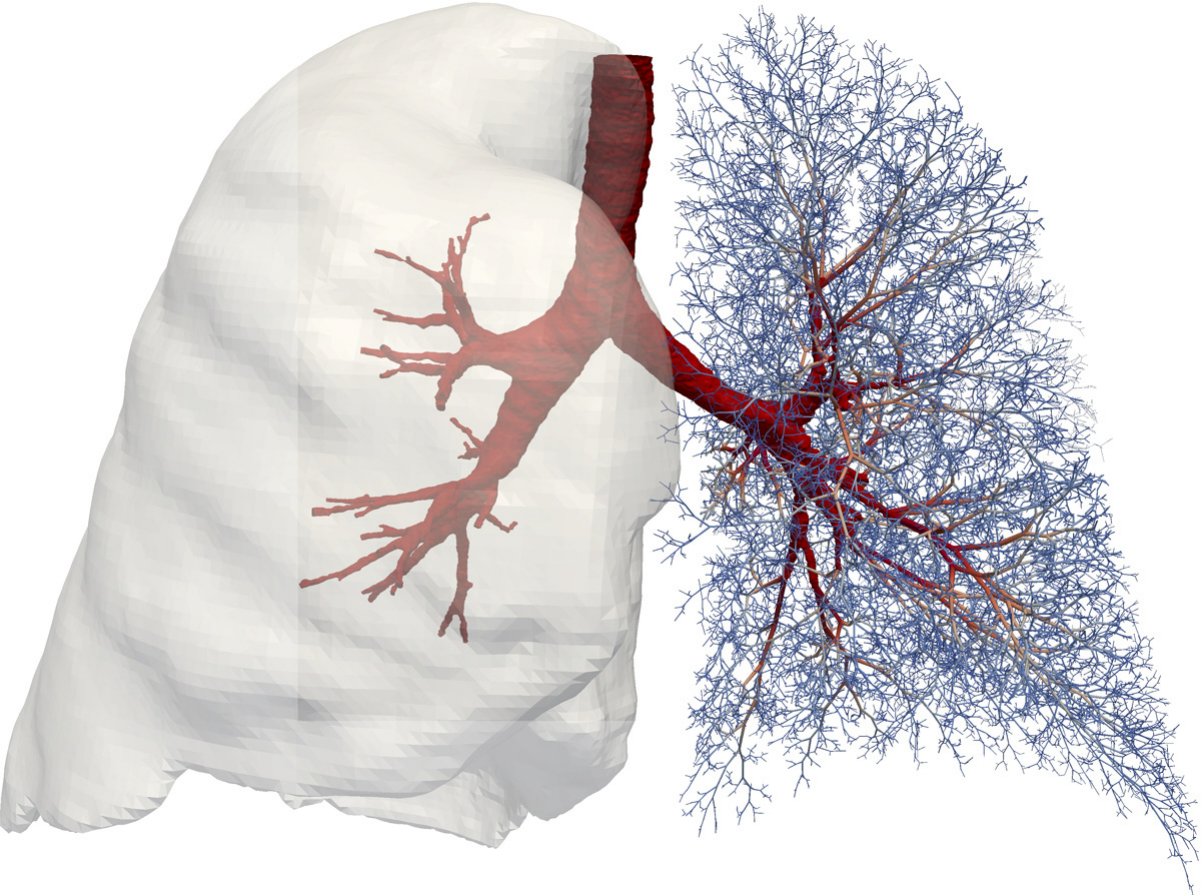
**Example of a patient-specific model of an AirPROM subject showing central airways (red) and lung surface (gray) segmented from CT and on the right the 1D volume-filling branching conducting airway network (Figure courtesy of Rafel Bordas, Oxford, **www.airprom.eu).

The main focus of the modeling within AirPROM is predicting ventilation and the impact of pathophysiological changes on resultant ventilation and lung function. The AirPROM workflow (Figure 3) includes data and models for both the large and small airways. State-of-the-art software (Mimics®, Materialise NV, Belgium; Airways, Institut Telecom, France) is being developed to enable automatic extraction of the morphological properties of central airways and lobes from patient CT data. High-resolution computational meshes of the central airways and lung surface are generated for use in 3D CFD (*FRI*) simulation studies using the ANSYS software. 1D airway models are generated down to the gas exchange level using the VFB algorithm (see Figure 4). Functional models that predict ventilation and impedance within the 1D networks have been developed. Correlation of model predictions with imaging and measures at the mouth will be used for model validation. At least 70 patient specific models will be analyzed in this pipeline. So far 24 patients within the AirPROM database have been analyzed using FRI, results have shown a good correlation with PFT measurements [54].

A combined HP MRI measurement and modeling approach is being used to assess the structure and function of the small airways [55]. In addition measurements of impedance and gas washout are being extracted from the AirPROM patients to provide information about the small airways. A micro-airway model is being developed, in parallel with an *ex vivo *tissue model, that incorporates tissue level mechanical models and cell level modeling techniques (using agent-based modeling). Statistical models are being used to capture the structure and function of the lung as a whole. All of these models - large and small airways, macro and micro scale - will be linked together to provide a multiscale modeling framework to predict the evolution of disease and response to clinical interventions [46].

## The challenges in moving forward

These types of modeling approaches suffer from several limitations. The process of modeling requires several simplifying assumptions of the system being modeled to enable feasibility of solution; models will never replicate the complexity of reality. However, a model need only be as complicated as required to answer a specific question. It is paramount that implications of the simplifying assumptions used are carefully considered when analyzing outcomes and applying models. A large part of the Physiome/VPH-related modeling drive aims towards creating 'patient-specific' models. However, only parts of a model can be personalized to a given patient with many unknowns remaining (such as tissue properties, structure of smaller airways/vessels, exact values of boundary conditions to use to name a few). In addition, the link between genetics and environment for an individual seems a near impossible feat to include in models. Current models typically predict function at a narrow time point of life. Predictions of the longer time scale evolution of disease will be a very difficult property to integrate into a modeling framework. At this stage, the power of modeling is predominantly in generating new hypotheses that can be tested experimentally. As new knowledge emerges models and modeling techniques improve models will hopefully move forward in the areas described above.

There is a pressing need for modelers and clinicians to work ever more closely to achieve the shared goal of patient-specific clinically relevant multiscale models. One of the major hurdles standing in the way of computational modeling and its clinical use is lack of model validation. Models need to be thoroughly tested to check the accuracy and reliability of the predictions before they will be accepted into the clinical environment. A recent paper by Pathmanathan and Gray [56] discusses the field of verification, validation and uncertainty quantification (VVUQ) and the need for this analysis to be applied to physiological models. This technique provides a methodical way of quantifying the accuracy (and therefore the uncertainty) in a model and will be vital in the clinical acceptance and usage of models.

## Conclusions

Here we have presented the current state-of-the-art techniques in image processing and computational modeling of the lung. A combined approach using imaging and modeling together provides an extremely powerful tool to assess features of lung (dys)function unable to be measured in the clinic. The AirPROM project aims to develop multiscale models of the lung. In comparison, the Synergy-COPD project aims at a systems approach - integrating models across different organ systems in the body. The work being conducted within the AirPROM project will complement Synergy-COPD by contributing models and knowledge of the function within the lung in health and during asthma and COPD.

## Competing interests

CB has received grant income and consultancy from GSK, AZ/MedImmune, Novartis, Chiesi, Roche/Genentech and BI. KSB and TD have no competing interests.

## Authors' contributions

KSB developed the plan for the manuscript and provided expertise and text on computational modeling aspects within the paper and the AirPROM project. TD provided expertise and text on image-based techniques. CB provided expertise in respiratory medicine and the AirPROM project. All authors were involved in editing the final manuscript.,'

## Declarations

Publication of this article has been funded by the European Union Seventh Framework Programme FP7/2007-2013 under grant agreement numbers 270086 (Synergy-COPD, http://www.synergy-copd.eu) and 270194 (AirPROM, http://www.airprom.eu). Data was provided with permission by Hospital Clinic, IDIBAPS, University of Barcelona.

This article has been published as part of *Journal of Translational Medicine *Volume 12 Supplement 2, 2014: Systems medicine in chronic diseases: COPD as a use case. The full contents of the supplement are available online at http://www.translational-medicine.com/supplements/12/S2.
